# Accuracy and limitations of ureteroscopic biopsy in the staging and grading of upper tract urothelial carcinoma: A retrospective analysis at a large tertiary center

**DOI:** 10.14440/bladder.2025.0006

**Published:** 2025-06-04

**Authors:** Tran Ngoc An Huynh, Xinyi Wei, Samiha Arulshankar, James Huang, Nieroshan Rajarubendra, Kevin Chu, Matthew Harper, Scott Donnellan, Weranja Ranasinghe

**Affiliations:** 1 Department of Urology, Monash Health, Melbourne, Victoria 3806, Australia; 2 Department of Surgery, School of Clinical Sciences at Monash Health, Monash University, Clayton, Victoria 3800, Australia

**Keywords:** Upper tract urothelial carcinoma, Ureteroscopy, Biopsy, Radical nephroureterectomy, Tumor staging, Tumor grading, Diagnostic accuracy, Renal pelvis tumors

## Abstract

**Background:**

Upper tract urothelial carcinoma (UTUC) poses significant diagnostic challenges due to the limitations of current staging and grading techniques. Ureteroscopic (URS) biopsy is widely used preoperatively, but its accuracy, compared to final pathology, remains variable.

**Objectives:**

To evaluate the accuracy and limitations of URS biopsy in the staging and grading of UTUC, using final pathological results from radical nephroureterectomy (RNU) as the reference standard.

**Methods:**

This retrospective study included 86 patients who underwent URS biopsy followed by RNU for UTUC at a tertiary institution between 2011 and 2023. Data were collected on patient demographics, tumor characteristics, imaging, and pathology results. The accuracy of URS biopsy in staging and grading was assessed, and its associations with pathological upstaging and non-diagnostic biopsies were statistically analyzed.

**Results:**

URS biopsy correctly staged 54.69% of tumors (κ = 0.311 [0.183 – 0.439], p<0.001) and correctly graded 70.93%. (κ = 0.447 [0.303 – 0.592], p<0.001). Pathological upstaging and upgrading occurred in 39.06% and 25.58% of cases, respectively. Non-diagnostic biopsies for both stage and grade were observed in 5.81% of cases, particularly in tumors located in the renal pelvis (p=0.0064). Complementary diagnostic tools, such as computed tomography urography (CTU) and urine cytology, showed limitations, with CTU detecting invasive disease in only 14.29% of cases and urine cytology identifying high-grade tumors in 11.11%.

**Conclusion:**

URS biopsy demonstrates limitations in accurately staging and grading UTUC, resulting in a risk of both undertreatment and overtreatment. A multimodal diagnostic approach incorporating imaging, cytology, and clinical judgment is essential to optimizing management decisions and improving oncological outcomes.

## 1. Introduction

Upper tract urothelial carcinoma (UTUC) is the malignant transformation of urothelial cells lining the pyelocaliceal cavities and ureter.^1^ In Western countries, the estimated annual incidence of UTUC stands at approximately two cases per 100,000 population, with incidence rates rising over the past three decades.^2^ This increase is likely due to advancements in diagnostic technologies and an aging population.^3^,^4^ Given the increase in diagnoses, accurate staging and grading of UTUC are essential for determining the most appropriate clinical management.

The diagnosis of UTUC typically involves a combination of urine cytology, computed tomography urography (CTU), and ureteroscopic (URS) biopsy.^5^ Urine cytology demonstrates higher sensitivity when selectively obtained from the affected upper tract; however, its sensitivity of 11 – 71% still makes it suboptimal for ruling out UTUC.^2^ CTU is considered the gold standard in radiological tumor detection for UTUC, with a sensitivity of 96% and specificity of 99%.^6^ While CTU provides accurate anatomical information, it has limited value in providing accurate information regarding tumor stage and grade.^7^ The definitive diagnosis and histopathological characterization largely rely on tissue sampling through URS biopsy. However, URS biopsy is associated with challenges such as insufficient and superficial tissue sampling, as well as specimen fragmentation during tissue processing, all of which can compromise the accuracy of both staging and grading.^8^ These inaccuracies are significant, as they may influence treatment decisions for both high-risk and low-risk diseases.

Current guidelines from the European Association of Urology (EAU) for UTUC recommend radical nephroureterectomy (RNU) as the standard treatment for high-risk UTUC. While RNU is effective for managing high-risk UTUC, it is associated with significant morbidity and perioperative complications. For patients with low-risk UTUC, nephron-sparing surgeries (NSS) have shown similar cancer-specific survival rates compared to RNU.^9^ Despite this, reluctance to pursue NSS still stems from concerns of undertreatment, often arising from inconsistencies between URS biopsy findings and actual pathological stage and grade. Therefore, accurate grading and staging are crucial for avoiding both overtreatment and undertreatment. Reliable diagnostics are essential for identifying patients who may benefit from NSS, minimizing morbidity while maintaining oncological safety. This study aimed to assess the accuracy of URS biopsy in the staging and grading of UTUC, using pathological results from RNU as the reference standard.

## 2. Materials and methods

This study was a retrospective analysis conducted at a multisite tertiary institution in Victoria, Australia. Records of all patients who had undergone RNU for UTUC between January 1, 2011, and December 31, 2023, were reviewed. The study was approved by the local institutional research ethics board (ethics committee name: Monash Health Human Research Ethics Committee; approval code: RES-23-0000-581Q).

Patients were eligible for inclusion if they were over 18 years of age and had undergone URS biopsy followed by RNU for localized UTUC. Localized disease was determined based on pre-operative imaging assessment. Patients were excluded if RNU was performed for non-UTUC-related reasons or if pathological records or clinical data were incomplete.

All cases were discussed at the departmental multidisciplinary tumor board (MDT) before surgery to establish treatment plans. Data were collected in accordance with the EAU risk stratification parameters for UTUC. The data included urine cytology, CTU findings regarding tumor invasion and pre-operative hydronephrosis, biopsy grade, tumor size, tumor multifocality, and the presence of variant histology. Additional information harvested included patient age, sex, and final pathological stage and grade as determined at RNU.

CTU was the primary imaging modality used for the pre-operative assessment of UTUC, providing detailed information on tumor size, hydronephrosis, and tumor staging. Tumor size was measured as the maximum diameter, either radiographically or endoscopically, if the lesion was too small to be visualized on imaging. Pre-operative clinical tumor staging was based on either URS biopsy or radiological findings, differentiating between organ-confined tumors (cTa – cT2) and locally invasive tumors (≥cT3). Multifocal tumors were defined as two or more tumors identified within the same upper urinary tract unit, either on imaging or visualization during URS. All pre-operative images were reviewed by a dedicated uroradiologist at MDT.

Histopathological assessment of URS biopsies and RNU specimens and cytological evaluation of urine samples were performed by institutional pathologists and reviewed by an uropathologist at the MDT.

Urine cytology was analyzed from either voided urine or selectively instrumented samples. The results were classified using the Paris System, which includes the following categories: Non-diagnostic, negative for high-grade urothelial carcinoma (NHGUC), atypical urothelial cells (AUC), suspicious for high-grade urothelial carcinoma (SHGUC), high-grade urothelial carcinoma (HGUC), and low-grade urothelial neoplasm (LGUN).

Tumor grading was based on the 2004/2016 World Health Organization/International Society of Urologic Pathologists consensus classification. Specimens demonstrating mixed high- and low-grade features were categorized as high-grade.

Clinical tumor staging for URS biopsy specimens was made on the basis of the level of invasion, ranging from cTa to cT4. Cases with absent subepithelial connective tissue due to superficial biopsy were classified as cTx.

RNU was performed using either an open or laparoscopic approach at the discretion of the operating surgeon. The choice of surgical technique was based on pre-operative imaging findings, intraoperative considerations, and MDT recommendations. In all cases, the procedure involved the removal of the kidney, the entire length of the ureter, and the bladder cuff, which was in line with the standard of care for UTUC.

URS biopsy stages and grades were compared to the final RNU pathology to determine diagnostic accuracy. Associations with pathological upstaging or unanalyzable biopsies were assessed using Fisher’s exact and Chi-square tests. The concordance between the biopsy and final stage and grade was evaluated using Cohen’s kappa. Multivariable logistic regression analysis was also performed to examine potential correlations between patient and tumor characteristics and pathological upstaging. Statistical analyses were performed using IBM SPSS v26 or GraphPad Prism software. A *p*<0.05 was considered statistically significant.

## 3. Results

**Figure 1:**
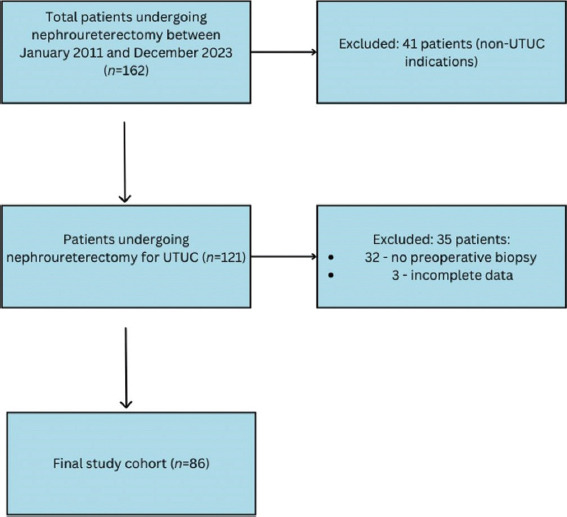
Flow chart of patient inclusion Abbreviation: UTUC: Upper tract urothelial carcinoma.

A total of 162 patients underwent RNU at our health service between January 1, 2011, and December 31, 2023. Patients who were <18 years old, did not undergo biopsy before RNU, had imaging identifying metastatic disease preoperatively, underwent RNU for non-UTUC-related causes or had incomplete data were excluded. Overall, 86 patients were included in the analysis (Figure 1).

The baseline characteristics of the patient cohort are summarized in Table 1. The median age was 71 years, ranging from 36 to 91 years. Of the patients, 70.93% were male and 19.07% were female. The mean number of biopsies was 2.69 (range: 1 – 8), and the average maximum biopsy dimension measured was 3.26 mm (range: 0.5 – 10 mm). On average, the interval from biopsy to nephroureterectomy was 66 days. Tumors were located in the ureter in 43.02% of cases, in the kidney in 39.53%, and in both regions in 8.14%. The mean tumor size was 38.32 mm (range: 1 – 215 mm). A comparative analysis of included and excluded patients who underwent RNU for UTUC was performed (Table A1), examining demographic and clinical characteristics.

**Table 1:** Baseline characteristics of the patient cohort

Patient characteristics	Value
Age (years)	71 (36–91)
Gender	
Male	61 (70.93)
Female	25 (29.07)
Tumor size (mm), mean (range)	38.32 (1 – 215)
Tumor location	
Ureter	43 (50)
Renal pelvis	35 (40.7)
Ureter and renal pelvis	8 (9.3)
Tumor grade at biopsy	
High	37 (43.02)
Low	39 (45.35)
Atypia	3 (3.48)
Unable to be assessed	7 (8.14)
Variant histology	11 (12.79)
Clinical tumor stage at biopsy	
cTx	22 (25.58)
cTa	48 (55.81)
cTis	1 (1.16)
cT1	13 (15.12)
cT2	2 (2.33)
Pre-operative cytology	
Negative	33 (38.37)
Atypical	16 (18.6)
Low grade	6 (6.98)
Suspicious for high-grade	3 (3.49)
High grade	1 (1.16)
Not available	27 (31.4)
Biopsy tool for ureteric tumors	
Forceps	14 (32.56)
Basket	11 (25.58)
Forceps and basket	2 (4.65)
Not documented	16 (37.21)
Biopsy tool for renal pelvis tumors	
Forceps	16 (45.71)
Basket	9 (25.71)
Forceps and Basket	1 (2.85)
Not documented	9 (25.71)
Biopsy tool for ureteric and renal pelvis tumors	
Forceps	6 (75)
Basket	0 (0)
Forceps and Basket	0 (0)
Not documented	2 (25)

Note: Data are presented as median (range) for continuous variables and as frequency (percentage) for categorical variables unless otherwise stated.

**Table A1:** Comparative analysis of excluded versus included patients who underwent radical nephroureterectomy for upper tract urothelial carcinoma

Patient characteristics	Excluded	Included	*p*-value
Age	71 (39 – 91)	71 (36 – 91)	0.44
Gender			
Male	16 (50)	61 (70.93)	0.06
Female	16 (50)	25 (29.07)	
Tumor size (mm), mean (range)	42.16 (10 – 95)	38.32 (1 – 215)	0.437
Variant histology	1 (3.12)	11 (12.79)	0.04*
Tumor location			
Ureter	7 (21.88)	43 (50)	0.004**
Kidney	18 (56.25)	35 (29.07)	
Ureter and kidney	7 (21.88)	8 (9.3)	

Notes: Data are presented as median (range) for continuous variables and as frequency (percentage) for categorical variables unless otherwise stated.

* *p*<0.05,

** *p*<0.01.

CTU demonstrated high accuracy in identifying tumor location, correctly localizing 87.8% of UTUC tumors when compared to the final RNU pathology. However, its ability to detect invasive disease was limited. Among patients with pT3 or higher-stage disease, CTU showed invasion in only 14.29% of cases, highlighting its limitations in assessing tumor depth and invasiveness.

Similarly, the diagnostic accuracy of urine cytology in identifying UTUC was suboptimal. Among 55 patients who underwent urine cytology, 33 (60%) yielded a negative result. Of the 36 patients with confirmed high-grade pathology who had urine cytology, only 4 (11.11%) were reported as HGUC or SHGUC. The remaining results were inconclusive or misclassified: 12 (33.33%) were categorized as AUC, 2 (5.56%) were classified as LGUN, and 18 (50%) were misidentified as NHGUC.

Among the URS biopsies taken before RNU, 55.81% were staged as cTa, 1.16% as cTis, 13.95% as cT1, and 2.33% as cT2. However, 26.74% of biopsies could not be assessed for tumor stage due to superficial or insufficient sampling. A statistically significant correlation was observed, with unanalyzable biopsy stages more frequently associated with renal tumors compared to ureteric tumors (*p*=0.0064).

The high-grade disease was identified in 43.02% of cases and low-grade disease in 45.35%, whereas 3.48% showed atypical cells without definitive grades. In 8.14% of cases, biopsy specimens were insufficient to assess tumor grade. A small subset (5.81%, *n*=6) of URS biopsies were non-diagnostic for both stage and grade due to insufficient material collected or fragmentation. Notably, all of these samples originated from tumors located in the renal pelvis; three were obtained using forceps, whereas the biopsy method was undocumented for the remaining three.

When comparing initial URS biopsy with final RNU results, tumor stage was correctly identified in 54.69% of cases (κ = 0.311 [0.183 – 0.439], *p*<0.001). Among those incorrectly staged, 86.21% had a higher stage in the final pathology. Overall, this led to a stage increase for 39.06% of the cohort (Table 2).

**Table 2:** Association between clinical stage from ureteroscopic biopsy and pathological stage from radical nephroureterectomy

Clinical stage (cT)	pT0/x	pTa	pTis	pT1	pT2	pT3	pT4	Downstaged (%)	Same stage (%)	Upstaged (%)	Total
cTa	2	32	0	4	5	5	0	2 (4.17)	32 (66.67)	14 (29.17)	48
cTis	0	0	0	1	0	0	0	-	-	1 (100)	1
cT1	0	1	1	3	0	7	1	2 (15.38)	3 (23.08)	8 (61.54)	13
cT2	0	0	0	0	0	1	1	-	-	2 (100)	2
Total	2	41	1	12	7	20	3	4 (6.25)	35 (54.69)	25 (39.06)	64

Notes: cT: Clinical tumor stage; pT: pathological tumor stage.

For tumor grading, URS biopsy accurately identified the grade in 70.93% of cases (κ = 0.447 [0.303 – 0.592], *p*<0.001). Among the misclassified cases, 88% were found to have a higher grade in the final pathology compared to the initial biopsy. This resulted in an overall grade increase of 25.58% of the cohort (Table 3).

**Table 3:** Association between ureteroscopic biopsy tumor grade and radical nephroureterectomy pathology

Ureteroscopic biopsy grade	Benign	Low grade	High grade	Down grade (%)	Same grade (%)	Up grade (%)	Total
Biopsy unclear/atypia	0	4	6	-	-	10 (100%)	10
Biopsy low-grade	2	25	12	2 (5.12)	25 (64.10)	12 (30.77)	39
Biopsy high-grade	0	1	36	1 (2.7)	36 (97.3)	-	37
Total	2	30	54	3 (3.49)	61 (70.93)	22 (25.58)	86

In ureteric tumors, biopsy accuracy was 78.57% for grade and 60.52% for stage. In renal tumors, the accuracy for grade and stage was 70% and 58.3%, respectively. For tumors involving both the ureter and kidney, accuracy was 62.5% for grade and 33% for stage.

Multivariable logistic regression analysis revealed no significant associations between pathological upstaging (Table 4) or upgrading (Table 5) and patient characteristics (*e.g*., age and sex), CTU findings (*e*.g., tumor invasion and hydronephrosis), or tumor-related factors (*e.g*., size, location, and variant histology).

**Table 4:** Multivariable analysis for pathological upstaging

Category	Odds ratio	Confidence interval	*p*-value
Age at surgery	1.667	0.995 – 1.154	0.0955
Female gender	1.204	0.5821 – 10.06	0.2285
Tumor size	1.551	0.9976 – 1.033	0.1209
Hydronephrosis	0.6928	0.4422 – 5.994	0.4884
Variant histology	1.520	0.7112 – 24.08	0.1284
Invasion on CTU	0.9294	0.2232 – 14.6	0.3527

Abbreviation: CTU: Computed tomography urography.

**Table 5:** Multivariable analysis for pathological upgrading

Category	Odds ratio	Confidence interval	*p*-value
Age at surgery	0.9431	0.8825 – 1.002	0.0635
Female gender	0.5697	0.1007 – 2.495	0.4902
Tumor size	1.010	0.9937 – 1.026	1.290
Hydronephrosis	0.8459	0.2052 – 3.505	0.8135
Variant Histology	1.305	0.1443 – 8.502	0.7909
Invasion on CTU	4.546	0.3607 – 68.44	0.2362

Abbreviation: CTU: Computed tomography urography.

## 4. Discussion

In our retrospective analysis, URS biopsy accurately staged 54.69% and graded 70.93% of cases in our cohort. A substantial proportion of patients experienced upstaging (39.06%) and upgrading (25.58%) at the final pathology, indicating the potential limitations of URS biopsy as a standalone tool for precise staging and grading. Accurate staging of UTUC is essential in guiding management, as it determines the extent of surgical intervention and the potential for nephron-sparing approaches. As the biopsy correctly staged fewer than half of the cases, with a significant portion of patients under-staged (54.65%), reliance on biopsy results alone could potentially result in undertreatment. Consistent with our findings, a large multi-institutional retrospective analysis by Mori *et al*.^10^ reported clinical understaging in 59.5% and undergrading in 42.4% of patients.

Moreover, the rates of non-diagnostic, unstaged, and ungraded biopsies in our study were 5.81%, 25.58%, and 11.6%, respectively. These results align with findings from the meta-analysis and systematic review by Subiela *et al.*,^11^ which reported rates of 8%, 32%, and 1%, respectively.

Several factors contribute to the limitations of URS biopsy in accurately staging and grading UTUC. The thinness of the upper urinary tract often discourages deeper sampling due to the risk of perforation and bleeding.^7^ In our cohort, a significant correlation was observed between unanalyzable biopsy specimens for staging and tumor presence in the renal pelvis (*p*=0.0064), suggesting that tumors in this region are more likely to yield suboptimal and superficial samples. This may be attributed to the challenging anatomical location and limited accessibility of the renal pelvis.^12^ In addition, renal pelvis tumors were smaller in size (median 30 mm, range 3 – 110 mm) and more often sampled using forceps rather than baskets (45.7% versus 25.7%). Baskets are known to produce larger samples; in our cohort, the average biopsy size obtained with a basket was 4.9 mm, compared to 2.5 mm for those harvested with forceps. Smaller biopsy samples may further compromise the adequacy of tissue for accurate histopathological evaluation.^13^,^14^

Complementary diagnostic modalities, such as CTU and urine cytology, also showed limitations. While CTU accurately identified tumor location in 87.8% of cases, its ability to detect invasive disease was poor. Among patients with ≥pT3 disease, CTU detected invasion in only 14.29% of cases. This low sensitivity may be ascribed to microscopic tumor invasion that falls below the resolution of imaging. Urine cytology similarly demonstrated poor diagnostic performance, with 60% of patients having negative results, and only 11.11% of patients with high-grade pathology were correctly identified as HGUG or SHGUC. Among patients who were understaged on biopsy, only two showed signs of invasion on CTU. Similarly, of those who were undergraded, only three had SHGUC on urine cytology; the remainder had negative or low-grade results. These findings highlight the limitations of CTU and urine cytology, even when used in conjunction with URS biopsy, underscoring the current challenges in pre-operative assessment.

Multivariable logistic regression analysis did not identify significant associations between pathological upstaging or upgrading and patient characteristics (age and sex), CTU evidence (tumor invasion or hydronephrosis), or tumor features (size, location, and variant histology). In contrast, the study by Mori *et al*.^10^ found that hydronephrosis, high-grade cytology, ureteric tumor location, female sex, and age were significantly associated with pathological upstaging in univariable analysis, with hydronephrosis being also significant in multivariable analysis.

Our study also identified overtreatment in two patients who were classified as low risk according to EAU risk stratification but were found to have low-grade non-invasive disease following RNU. These cases highlight the possibility of overtreatment when tumor risk is overestimated.

The findings of this study underline the limitations of URS biopsy in providing precise staging and grading of UTUC, which in turn impedes the feasibility of kidney-sparing management due to high rates of understaging and undergrading. Given these limitations, clinicians should exercise caution when interpreting URS biopsy results in isolation. Multidisciplinary discussions that incorporate imaging, cytology, and clinical suspicion are essential for optimizing decision-making and mitigating the risks of both undertreatment and overtreatment. Moreover, as tumors located in the renal pelvis are significantly associated with higher rates of unstaged biopsies, likely due to the anatomical challenges of this region, clinicians should be especially cautious in such cases. Additional diagnostic strategies, improved tissue preservation techniques, or a lower threshold for more aggressive intervention may be warranted.

The difficulty in obtaining adequate samples during URS biopsy may be addressed through the use of appropriate biopsy techniques and devices. A newer URS biopsy method termed the “form tackle” technique and described by Klett *et al*.^15^ involves advancing the ureteroscope 3 – 10 mm after forceps closure, then withdrawing it from the patient. This technique has been shown to yield larger tissue samples. Furthermore, new specimen-handling techniques aimed at maximizing tissue preservation can also be adopted. A study by Golan *et al*.^16^ examined the effectiveness of a novel technique in improving the histopathological yield of small URS biopsies. With this method, the specimen is mounted on filter paper before paraffin embedding. The study found that this technique significantly increased the proportion of biopsies in which tumor stage could be determined, from 25% using the standard method to 63% using the filter paper technique (*p*=0.007). Stage concordance with the final RNU pathology was also higher using this method (56% versus 27%). These findings support the potential benefits of adopting innovative tissue handling and biopsy techniques to improve the diagnostic yield and accuracy of URS biopsies.

Emerging diagnostic approaches such as magnetic resonance urography (MRU) and photodynamic diagnosis (PDD) may help address some limitations of current pre-operative assessments of UTUC. Notably, a prospective study by Messina *et al*.^17^ reported that MRU achieved a sensitivity of 95% and specificity of 71% in diagnosing muscle-invasive disease. Meanwhile, a systematic review and meta-analysis by Liu *et al*.^18^ demonstrated that PDD could distinguish UTUC from non-cancerous urothelium, with a sensitivity of 0.96 and specificity of 0.86. These findings suggest that incorporating MRU and PDD into the pre-operative assessment of UTUC could improve both lesion detection and characterization, particularly in cases where traditional imaging or biopsy proves inadequate.

This study has several limitations. First, its retrospective design and the exclusion of patients who did not undergo RNU likely introduced a selection bias toward higher-grade and more invasive tumors. Second, the single-institution setting and relatively small sample size may limit the generalizability of the findings. Moreover, both biopsy and RNU procedures in this study were not standardized and were performed by multiple surgeons, increasing procedural variability. Finally, histopathological assessments were conducted by different pathologists at the institution, which may have introduced interobserver variability in the grading and staging of biopsy specimens. To better understand potential biases, the baseline characteristics of excluded patients were compared with those of patients included in the analysis (Table A1). While most variables were comparable, notable differences were observed in the proportion of variant histology and tumor location. Future prospective, multicenter studies with standardized biopsy and RNU protocols are required to validate our findings and further clarify the factors affecting the accuracy of URS biopsy in UTUC grading and staging.

## 5. Conclusion

There is significant discordance between the stage and grade-determined URS biopsy and those found in final RNU pathology, with a clear tendency toward understaging and undergrading. In addition, a substantial proportion of biopsies were non-diagnostic or unstageable, particularly in renal pelvis tumors, underscoring the challenges posed by anatomical constraints and limited specimen quality.

These results emphasize the importance of a multimodal approach to staging and grading of UTUC before RNU, integrating imaging, urine cytology, and clinical judgment to complement URS biopsy results. Sole reliance on URS biopsy may lead to disease misclassification and inappropriate treatment strategies, potentially resulting in either overtreatment or undertreatment.

## Data Availability

The dataset used and analyzed in the current study may be available from the corresponding author upon reasonable request and with approval from the relevant ethics committee.
